# Hierarchical structural complexity in atomically precise nanocluster frameworks

**DOI:** 10.1093/nsr/nwaa077

**Published:** 2020-04-24

**Authors:** Xiao Wei, Xi Kang, Zewen Zuo, Fengqi Song, Shuxin Wang, Manzhou Zhu

**Affiliations:** Department of Chemistry and Centre for Atomic Engineering of Advanced Materials, Anhui Province Key Laboratory of Chemistry for Inorganic/Organic Hybrid Functionalized Materials, Anhui University, Hefei 230601, China; Key Laboratory of Structure and Functional Regulation of Hybrid Materials (Anhui University), Ministry of Education, Hefei 230601, China; Department of Chemistry and Centre for Atomic Engineering of Advanced Materials, Anhui Province Key Laboratory of Chemistry for Inorganic/Organic Hybrid Functionalized Materials, Anhui University, Hefei 230601, China; Key Laboratory of Structure and Functional Regulation of Hybrid Materials (Anhui University), Ministry of Education, Hefei 230601, China; National Laboratory of Solid State Microstructures, Collaborative Innovation Center of Advanced Microstructures, School of Physics, Nanjing University, Nanjing 210093, China; Atomic Manufacture Institute, Nanjing 211805, China; National Laboratory of Solid State Microstructures, Collaborative Innovation Center of Advanced Microstructures, School of Physics, Nanjing University, Nanjing 210093, China; Atomic Manufacture Institute, Nanjing 211805, China; Department of Chemistry and Centre for Atomic Engineering of Advanced Materials, Anhui Province Key Laboratory of Chemistry for Inorganic/Organic Hybrid Functionalized Materials, Anhui University, Hefei 230601, China; Key Laboratory of Structure and Functional Regulation of Hybrid Materials (Anhui University), Ministry of Education, Hefei 230601, China; Department of Chemistry and Centre for Atomic Engineering of Advanced Materials, Anhui Province Key Laboratory of Chemistry for Inorganic/Organic Hybrid Functionalized Materials, Anhui University, Hefei 230601, China; Key Laboratory of Structure and Functional Regulation of Hybrid Materials (Anhui University), Ministry of Education, Hefei 230601, China

**Keywords:** hierarchical structural complexity, atomically precise nanocluster, 1D linear chain, 2D grid network, 3D superstructure

## Abstract

The supramolecular chemistry of nanoclusters is a flourishing area of nano-research; however, the controllable assembly of cluster nano-building blocks in different arrays remains challenging. In this work, we report the hierarchical structural complexity of atomically precise nanoclusters in micrometric linear chains (1D array), grid networks (2D array) and superstructures (3D array). In the crystal lattice, the Ag_29_(SSR)_12_(PPh_3_)_4_ nanoclusters can be viewed as unassembled cluster dots (**Ag_29_–0D**). In the presence of Cs^+^ cations, the Ag_29_(SSR)_12_ nano-building blocks are selectively assembled into distinct arrays with different oxygen-carrying solvent molecules―Cs@Ag_29_(SSR)_12_(DMF)*_x_* as 1D linear chains (**Ag_29_–1D**), Cs@Ag_29_(SSR)_12_(NMP)*_x_* as 2D grid networks (**Ag_29_–2D**), and Cs@Ag_29_(SSR)_12_(TMS)*_x_* as 3D superstructures (**Ag_29_–3D**). Such self-assemblies of these Ag_29_(SSR)_12_ units have not only been observed in their crystalline state, but also in their amorphous state. Due to the diverse surface structures and crystalline packing modes, these Ag_29_-based assemblies manifest distinguishable optical absorptions and emissions in both solutions and crystallized films. Furthermore, the surface areas of the nanocluster crystals are evaluated, the maximum value of which occurs when the cluster nano-building blocks are assembled into 2D arrays (i.e. **Ag_29_–2D**). Overall, this work presents an exciting example of the hierarchical assembly of atomically precise nanoclusters by simply controlling the adsorbed molecules on the cluster surface.

## Teaser

Micrometric linear chains (1D array), grid networks (2D array), and superstructures (3D array) of nanoclusters were selectively constructed by hierarchical self-assembly of Ag_29_(SSR)_12_ nano-building blocks with different solvent-conjoining Cs^+^ cations.

## INTRODUCTION

The past two decades have witnessed significant research efforts on atomically precise metal nanoclusters [1–26]. Amongst the nanocluster science, the self-assembly of cluster building blocks has been the subject of an intense investigation to achieve a wide range of multi-dimensional nanomaterials with ordered architectures [27–39]. Such assemblies originate in different types of inter-cluster interactions such as chemical bonding, hydrogen bonding, electrostatic, van der Waals, *π*···*π* and C-H···*π* interactions [27,28]. On one hand, these cluster-based aggregates typically display enhanced performance (e.g. stability and fluorescence) relative to their constituent cluster building blocks owing to the synergy from the cluster−linker−cluster assembly system [27–39]. On the other hand, the precise structures of nanoclusters allow for the atomic-level understanding of inter-cluster interaction modes, and such knowledge further guides us to controllably constitute assembled cluster-based nanomaterials [27–39].

In general, nanocluster building blocks are assembled *via* the introduction of inter-cluster linkers (e.g. sulfur/nitrogen-carrying, multi-dentate molecules)―the covalent interactions between sulfur/nitrogen terminals of linkers and the Au/Ag surface atoms of nanoclusters are exploited to motivate the inter-cluster assembly [40–47]. For instance, by altering the bidentate nitrogenous linkers, Zang and co-workers constructed a series of 1D-to-3D Ag_14_ cluster-assembled nanomaterials [40]. Lei *et al*. presented the self-assembly of Ag_6_Au_6_ clusters by forming both inward and outward Ag-N interactions [45]. In both cases, the assembled modes can be dictated by the control over the nitrogen-carrying linkers [40–45].

Most recently, we have proposed a novel cluster-assembly pattern, namely, capturing Cs^+^ cations and dimethylformamide (DMF) molecules onto the nanocluster surface [48]. Specifically, in the crystal lattice, the Cs^+^−DMF−cluster interactions assemble the Ag_29_(SSR)_12_ nano-building blocks into 1D linear chains (SSR = 1,3-benzene dithiol) [48]. Considering that such an assembly largely relies on the Cs^+^−O interactions (the O junction site comes from the DMF), we perceive a good opportunity to control the assembly modes of Ag_29_(SSR)_12_―simply altering the oxygen-carrying solvents in the crystallization.

**Scheme 1.**
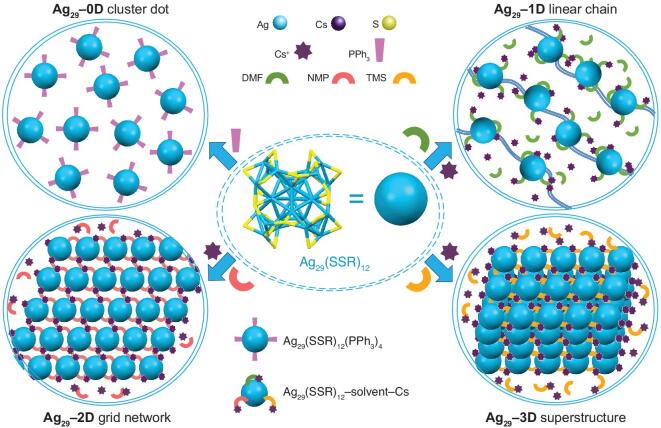
Scheme illustration of the 1D–3D assemblies of Ag_29_(SSR)_12_ nano-building blocks―including **Ag_29_–0D** cluster dots in the presence of PPh_3_, **Ag_29_–1D** linear chains (1D array) in the presence of Cs^+^ and DMF, **Ag_29_–2D** grid networks (2D array) in the presence of Cs^+^ and NMP, and **Ag_29_–3D** superstructures (3D array) in the presence of Cs^+^ and TMS.

Herein, the Ag_29_(SSR)_12_ nanocluster building blocks are selectively assembled into micrometric linear chains (1D array), grid networks (2D array) and superstructures (3D array), and such hierarchical constructions are determined by the single crystal X-ray diffraction (SC-XRD). Specifically, the presence of PPh_3_ (or the absence of Cs^+^) yields unassembled cluster dots (Ag_29_(SSR)_12_(PPh_3_)_4_, **Ag_29_–0D**). By comparison, when the Cs^+^ cations are captured on the nanocluster surface with different oxygen-carrying solvent molecules, the Ag_29_(SSR)_12_ nano-building blocks are selectively assembled into distinct arrays (Scheme 1)―the capture of Cs^+^-DMF on Ag_29_(SSR)_12_ producing 1D linear chains (Cs@Ag_29_(SSR)_12_(DMF)*_x_*, **Ag_29_–1D**), the capture of Cs^+^-NMP on Ag_29_(SSR)_12_ making up 2D grid networks (Cs@Ag_29_(SSR)_12_(NMP)*_x_*, **Ag_29_–2D**; NMP = N-methyl-2-pyrrolidone), and the capture of Cs^+^-TMS giving rise to 3D superstructures (Cs@Ag_29_(SSR)_12_(TMS)*_x_*, **Ag_29_–3D**; TMS = tetramethylene sulfone). Besides, the 1D–3D assemblies of these Ag_29_(SSR)_12_ nano-building blocks have not only been observed in their crystalline state, but also in their amorphous state, with the help of the aberration-corrected high angle annular dark field scanning transmission electron microscope (HAADF-STEM). Furthermore, the optional assembly modes result from different cluster–Cs–solvent interactions. Because of the different surface structures and crystalline packing modes, these Ag_29_-based assemblies manifest distinguishable optical absorptions and emissions in both solutions and crystallized films. Moreover, the surface areas and pore size distributions of the crystals of these nanoclusters are evaluated, and the maximum value of the surface area is reached when the cluster nano-building blocks are assembled into 2D arrays (i.e. **Ag_29_–2D**).

## RESULTS AND DISCUSSION

### Ag_29_–0D cluster dot and Ag_29_–1D linear chain

**Figure 1.**
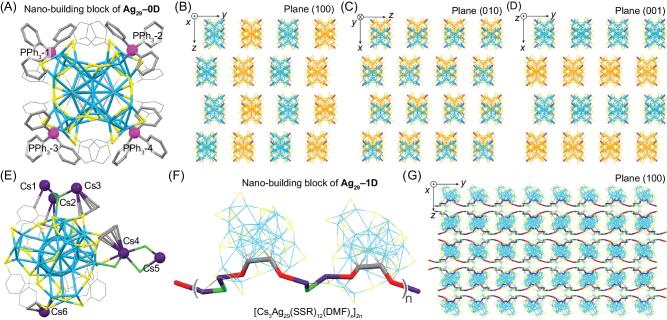
Crystal structures and crystalline packing modes of **Ag_29_–0D** and **Ag_29_–1D**. (A) Crystal structure (nano-building block) of **Ag_29_–0D**. (B–D) Packing of **Ag_29_–0D** in the crystal lattice: view from the *x* axis (B), *y* axis (C) and *z* axis (D). The Ag_29_ entities in different colors represent their locations in different layers in the crystal lattice. (E) Crystal structure of **Ag_29_–1D**. (F) Nano-building block of **Ag_29_–1D**. The two Ag_29_ cluster units are in differently twisting angles. (G) Packing of **Ag_29_–1D** in the crystal lattice, viewed from the plane (100). Color codes: light blue/orange sphere/stick, Ag; yellow/red sphere/stick, S; magenta sphere/stick, P; dark purple sphere/stick, Cs; green sphere/stick, O. For clarity, all H, N atoms, some C, Cs^+^ atoms and DMF molecules are omitted. Each green atom (O) represents a DMF molecule.

The Ag_29_(SSR)_12_ framework is composed of an icosahedral Ag_13_ kernel that is stabilized by an Ag_12_(SSR)_12_ shell, and the obtained Ag_25_(SSR)_12_ structure is further capped by four bare Ag atoms with a tetrahedral pattern (Fig. S1) [49,50]. Although the Ag_29_(SSR)_12_ compound could exist in isolation, its four Ag terminals have a strong disposition to be sealed by the introduced PPh_3_ ligand, giving rise to the Ag_29_(SSR)_12_(PPh_3_)_4_ (**Ag_29_–0D**) nanocluster (Figs 1A and S2A). In the crystal lattice, all **Ag_29_–0D** entities are independent without any direct inter-cluster interactions in either direction (Fig. 1B–D). Accordingly, the presence of PPh_3_ with Ag_29_(SSR)_12_ yields the unassembled cluster dots, representing the zero-dimensional arrangement of the Ag_29_ cluster entities in the crystalline cell.

The capture of Cs^+^ cations with **Ag_29_–0D** dissociates the PPh_3_ ligands from the nanocluster surface, giving rise to Cs@Ag_29_(SSR)_12_(DMF)*_x_* (**Ag_29_–1D**, as depicted in Figs 1E and S2B) [48]. Besides, the interactions among the cluster framework, the Cs^+^ cations, and the DMF molecules assemble the Ag_29_(SSR)_12_ nano-building blocks into

cluster-based linear chains (Fig. 1F and G). As shown in Figs 1G and S3, the Ag_29_-based, 1D linear chains extend along the *y* axis, and the inter-chain distance between two adjacent cluster lines along the *z* direction is 17.291 Å [48]. Collectively, the introduction of Cs^+^ cations and DMF molecules onto the Ag_29_ nanocluster surface assembles the cluster dots into linear arrays, representing the 1D arrangement of the Ag_29_ cluster entities in the crystalline cell.

### Ag_29_–2D grid networks

**Figure 2.**
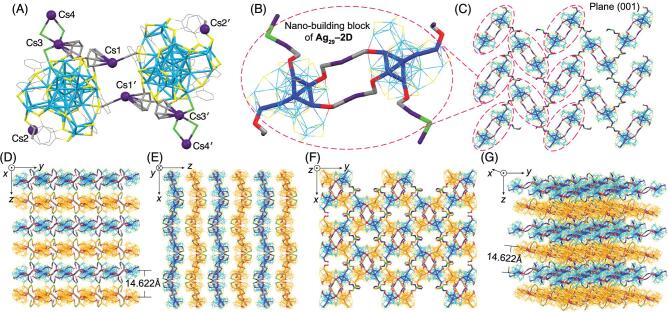
Crystal structure and crystalline packing mode of **Ag_29_–2D**. (A, B) Crystal structure (nano-building block) of **Ag_29_–2D**. The two adjacent Ag_29_ cluster units are in differently twisting angles. (B) is the enlargement of the circled section in (C). (C) Packing of **Ag_29_–2D** grid network in the crystal lattice, viewed from the plane (001). (D–F) Packing of **Ag_29_–2D** in the crystal lattice: view from the *x* axis (D), *y* axis (E) and *z* axis (F). (G) Packing of **Ag_29_–2D** in the crystal lattice from the *x* axis with a certain rotation, for observing the assembled grid networks more intuitively. As depicted in (D, G), the inter-layer distance is 14.622 Å. Color codes: light blue/orange/blue sphere/stick, Ag; yellow/red sphere/stick, S; grey sphere/stick, C; green sphere/stick, O; dark purple sphere/stick, Cs. For clarity, all H, N atoms, some C, Cs^+^ atoms and NMP molecules are omitted. Each green atom (O) represents an NMP molecule.

Considering that the aforementioned 1D assembly largely relies upon the Cs^+^−O interactions where the oxygen junction site comes from the DMF, we perceive a good opportunity to tailor the assembled modes of Ag_29_(SSR)_12_ nano-building blocks―altering the oxygen-carrying solvents in the crystallization. We first replaced DMF molecules in **Ag_29_–1D** into NMP to produce the Cs@Ag_29_(SSR)_12_(NMP)*_x_* (**Ag_29_–2D**; see the Methods Section for the detailed preparation). Significantly, the 2D-array assembly of Ag_29_ cluster entities was accomplished in the crystal lattice (Fig. 2). Structurally, the nano-building block of **Ag_29_–2D** contains two Ag_29_(SSR)_12_ compounds, six Cs^+^ cations, and several NMP molecules (Figs 2A, B and S2C). The two Ag_29_(SSR)_12_ compounds are in different twisting angles, and are mutually connected by two Cs^+^ cations (Cs1 and Cs1^′^) through Cs–C and Cs–*π* interactions (Fig. 2A and B). In addition, the inter-cluster assembly is induced by the outward interactions from four Cs^+^ conjunction sites―Cs2, Cs2^′^, Cs4 and Cs4^′^. The Cs4 is bonded on the nanocluster surface through Cs4-NMP-Cs3-cluster interactions (the same to Cs4^′^), whereas the Cs2 is directly anchored onto the nanocluster surface by Cs–C interactions (the same to Cs2^′^). Of note, the Cs4 (or Cs4^′^) on one cluster nano-building block also acts as the Cs2 (or Cs2^′^) of the adjacent block. In this context, the number of Cs^+^ cations in each nano-building block is six, and the ratio between [Ag_29_(SSR)_12_]^3−^ and Cs^+^ is exactly 1:3, for achieving the charge balance (Fig. 2A and B).

**Figure 3.**
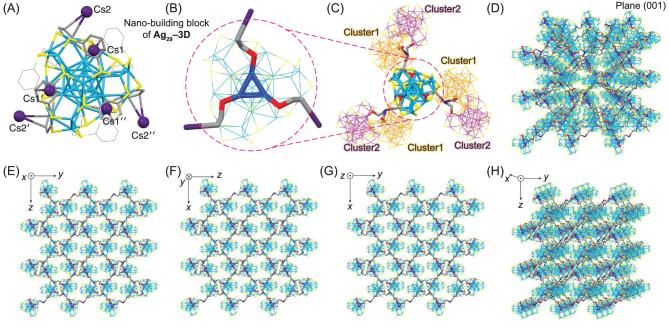
Crystal structure and crystalline packing mode of **Ag_29_–3D**. (A, B) Crystal structure (nano-building block) of **Ag_29_–3D**. (B) is the enlargement of the circled section in (C). Each **Ag_29_–3D** cluster is surrounded by six adjacent **Ag_29_–3D** nanoclusters including three cluster1 and three cluster2, giving rise to the 3D superstructure of Ag_29_ nano-building blocks. (D) Packing of the **Ag_29_–3D** superstructure in the crystal lattice, viewed from the plane (001). (E–G) Packing of **Ag_29_–3D** in the crystal lattice: view from the *x* axis (E), *y* axis (F) and *z* axis (G). (H) Packing of **Ag_29_–3D** in the crystal lattice from the *x* axis with a certain rotation, for observing the assembled superstructure more intuitively. Color codes: light blue/blue/orange/magenta sphere/stick, Ag; yellow/red sphere/stick, S; grey sphere/stick, C; dark purple sphere/stick, Cs. For clarity, all H, O, N atoms, some C, Cs^+^ atoms and TMS molecules are omitted. Each green atom (O) represents a TMS molecule.

For the 2D-array assembly, each [Cs@Ag_29_(SSR)_12_(NMP)*_x_*]_2_ unit is adjacent to four identical units through the four Cs^+^ conjunction sites, making up an Ag_29_-based, two-dimensional grid network (Fig. 3B and C). The grid network extends along the (001) plane, or both *x* and *y* axes (Fig. 3C–G). Along the *z* direction, the two neighboring networks display no interaction, but are in a face-symmetric relationship (the two types of layers are labeled in blue and orange of Ag atoms in Fig. 3D–G). In this context, the assembly of **Ag_29_–2D** in the crystal lattice follows an ABAB layer-by-layer packing mode.

The inter-layer distance (from kernel Ag to kernel Ag, as shown in Fig. 2D and G) between two adjacent networks is 14.622 Å. Overall, the capture of Cs^+^ and NMP of the Ag_29_(SSR)_12_ framework enables the self-assembly of cluster dots into grid networks, representing the two-dimensional arrangement of the Ag_29_ nano-building blocks in the crystalline cell.

### Ag_29_–3D superstructure

The further substitution of oxygen-carrying solvent molecules (DMF of **Ag_29_–1D**, or NMP of **Ag_29_–2D**) into TMS yields Cs@Ag_29_(SSR)_12_(TMS)*_x_* (**Ag_29_–3D**; see the Methods Section for the detailed preparation), which follows a 3D-array assembly in the crystal lattice (Figs 3 and S2D). To the nano-building block of **Ag_29_–3D**, all Cs^+^ cations are directly anchored onto the nanocluster surface through Cs–C interactions (Fig. 3A). For each **Ag_29_–3D** nano-building block, there are six Cs^+^ conjunction sites that are subordinate to two categories: inward Cs1, Cs1^′^ and Cs1^″^ that are simply bonded on the nanocluster surface, and outward Cs2, Cs2^′^ and Cs2^″^ that induce the inter-cluster assembly (Fig. 3A and B).

For the 3D-array assembly (Fig. 3C), each **Ag_29_–3D** nano-building block is surrounded by six adjacent nanoclusters including three cluster1 (labeled in orange of Ag atoms) and three cluster2 (labeled in magenta of Ag atoms). More specifically, each outward Cs^+^ cation connects one cluster1 and one cluster2, of which the cluster1 is arranged downwardly but the cluster2 is organized upwardly, constructing the Ag_29_-based, three-dimensional superstructures (Fig. 3C and D). The **Ag_29_–3D** nano-building blocks are assembled with a cubic pattern in the crystal lattice (i.e. a = b = c, and α = β = γ for the cell parameter). In this context, the cluster packing modes are identical in all directions, making up a highly symmetrical superstructure (Fig. 3E–H). Taken together, the bonding of Cs^+^ and TMS on Ag_29_(SSR)_12_ triggers the self-assembly of cluster dots into superstructures, representing the three-dimensional arrangement of the Ag_29_ nano-building blocks in the crystalline cell.

### Comparison of crystal structures and packing modes

Due to the different surfaces, these Ag_29_ nanoclusters (**Ag_29_–0D**, **Ag_29_–1D**, **Ag_29_–2D**, and **Ag_29_–3D**) exhibited distinct crystal structures and crystalline packing modes (Figs S4 and S5, and Tables S1 and S2). Although the overall Ag_29_(SSR)_12_ configuration retained from **Ag_29_–0D** to **Ag_29_–1D**,

**Ag_29_–2D** and **Ag_29_–3D**, obvious changes have been observed by comparing the corresponding bond lengths. Specifically, all of the three types of Ag-Ag interactions (Ag(core)–Ag(icosahedral shell), Ag(icosahedral shell)–Ag(icosahedral shell), and prism-like Ag(icosahedral shell)–Ag(motif) bonds) in Cs@Ag_29_ nanoclusters (**Ag_29_–1D**, **Ag_29_–2D**, and **Ag_29_–3D**) were much longer than those in the PPh_3_@Ag_29_ nanocluster (**Ag_29_–0D**), demonstrating an expanding trend of the overall framework along with the PPh_3_ dissociated process (Fig. S4A–C and Table S1). For the pyramid-like interactions between the vertex Ag and the icosahedral Ag, the bond lengths were all close to 3.04 Å for Cs@Ag_29_ nanoclusters. However, no analogous interaction was observed in the PPh_3_@Ag_29_ nanocluster since the corresponding distances ranged from 3.493 to 3.643 Å (Fig. S4D and Table S1). In this context, the vertex Ag atoms became closer to the icosahedral kernel when the **Ag_29_–0D** nanocluster was transformed into **Ag_29_–1D**, **Ag_29_–2D** and **Ag_29_–3D**, and the newly generated Ag_4_ pyramids were anticipated to make the Ag_29_(SSR)_12_ framework more robust.

The chemical environments of Cs^+^ ions in different Ag_29_-based assemblies have been compared. For **Ag_29_–1D**, three Cs^+^ ions (Cs1, Cs2 and Cs3) stabilize the cluster surface and the other three Cs^+^ ions (Cs4, Cs5 and Cs6) assemble Ag_29_ nano-building blocks into 1D linear chains (Fig. 1). For **Ag_29_–2D**, all Cs^+^ ions are used to activate the assembly of cluster nano-building blocks into 2D grid networks (Fig. 2). For **Ag_29_–3D**, the inward Cs^+^ ions (Cs1, Cs1^′^ and Cs1^″^) stabilize the cluster surface, and the outward Cs^+^ ions (Cs2, Cs2^′^ and Cs2^″^) induce the inter-cluster assembly of cluster nano-building blocks into 3D superstructures (Fig. 3). Of note, only the presence of Cs^+^ can induce the assembly of Ag_29_ nano-building blocks; by comparison, the **Ag_29_–0D** nanocluster maintains its structure in the presence of Li^+^, Na^+^ or K^+^ cations [48].

The crystalline packing modes of these Ag_29_-based assemblies were further compared (Fig. S5 and Table S2). Of note, two types of crystallization patterns of **Ag_29_–0D** have been reported―**Ag_29_–0D-cubic** and **Ag_29_–0D-trigonal**―due to their different crystallization processes [49,50]. Because of the distinct interactions among Ag_29_ clusters, Cs^+^ cations and solvent molecules, **Ag_29_–1D**, **Ag_29_–2D** and **Ag_29_–3D** were also crystallized in different systems. Specifically, although both **Ag_29_–1D** and **Ag_29_–2D** follow an orthorhombic packing mode, their unit cell parameters (i.e. values of a, b, c) were totally different (Table S2). The **Ag_29_–3D** displayed a cubic packing mode, the same as that of **Ag_29_–0D-cubic**, whereas the unit size of **Ag_29_–3D** was remarkably smaller than the **Ag_29_–0D-cubic** (14375 Å^3^ versus 40006 Å^3^; see details in Table S2). Such differences reflected both the molecular effects of the Cs^+^ capture and the solvent effects in affecting nanocluster geometric structures and crystalline packing patterns.

Notably, the hierarchically 1D-, 2D- and 3D-array assemblies of Ag_29_ building blocks have not only been observed in their crystalline state, but also in their amorphous state. Specifically, the aberration-corrected HAADF-STEM images of **Ag_29_–0D**, **Ag_29_–1D**, **Ag_29_–2D** and **Ag_29_–3D** nanoclusters were obtained by recording the drying solutions of these nanoclusters on carbon films. The **Ag_29_–0D** cluster entities were still discrete under the microscope vision (Fig. S6A), whereas some linear assembled **Ag_29_–1D** clusters were discovered (Fig. S6B). Given that **Ag_29_–0D** and **Ag_29_–1D** were controlled to the same concentration in the aberration-corrected HAADF-STEM detection, the 1D-array assembly of **Ag_29_–1D** indeed existed in its non-crystalline state. Figure S6C and D exhibited the HAADF-STEM images of **Ag_29_–2D** and **Ag_29_–3D**. Of note, for promoting the 2D-array and 3D-array assemblies of these two nanoclusters, the concentrations of them were much higher than in **Ag_29_–0D** and **Ag_29_–1D**. Compared with **Ag_29_–1D**, which displayed the linear assembly, the **Ag_29_–2D** nano-building blocks were more inclined to be aggregated with a 2D-array reticular pattern (Fig. S6C). Furthermore, although most cluster entities were discrete in the HAADF-STEM image of **Ag_29_–3D**, several cluster-based, 3D aggregates have been observed (Fig. S6D), which unambiguously demonstrated the 3D-array assembly of some Ag_29_(SSR)_12_ cluster entities. To sum up, the introduction of Cs^+^ cations and oxygen-carrying solvents was also able to induce the self-assembly of Ag_29_(SSR)_12_ nano-building blocks in the non-crystalline state.

### Characterization of Ag_29_-based assemblies

The electrospray ionization mass spectrometry (ESI-MS) measurement was firstly performed to verify the specific composition of each nanocluster (Fig. S7). Mass spectra of **Ag_29_–0D** showed five peaks that corresponded to [Ag_29_(SSR)_12_(PPh_3_)_4_]^3−^, [Ag_29_(SSR)_12_(PPh_3_)_3_]^3−^, [Ag_29_(SSR)_12_(PPh_3_)_2_]^3−^, [Ag_29_(SSR)_12_(PPh_3_)_1_]^3−^ and [Ag_29_(SSR)_12_]^3−^, respectively, in good agreement with the reported ‘dissociation-aggregation pattern’ of the PPh_3_ ligands on the **Ag_29_–0D** surface (Fig. S7A) [51]. These PPh_3_-containing signals were absent in the spectra of Cs@Ag_29_ nanoclusters because the PPh_3_ ligands had been dissociated from the nanocluster surface induced by the Cs^+^ capture. Two intense peaks, matching with the [Ag_29_(SSR)_12_]^3−^ and [CsAg_29_(SSR)_12_]^2−^ compounds, were observed for each mass spectrum of **Ag_29_–1D**, **Ag_29_–2D** or **Ag_29_–3D** (Fig. S7B–D), which verified the Cs^+^ capture in these nanoclusters. However, as to the mass spectra of each Cs@Ag_29_ nanocluster, only the single Cs^+^-adhered Ag_29_ compound (i.e. CsAg_29_(SSR)_12_) could be detected. The unattained mass signals of the complete Cs@Ag_29_ molecules resulted from the weak interactions among the Ag_29_(SSR)_12_ frameworks, the Cs^+^ cations, and the solvent molecules when the nanoclusters were in solutions.

^133^Cs and ^31^P nuclear magnetic resonance (NMR) were then recorded to validate the capture of PPh_3_ ligands or Cs^+^ ions on the Ag_29_ nanocluster surface. As depicted in Fig. S8A, the ^133^Cs NMR of CH_3_COOCs showed an intense signal at 72.74 ppm, and this signal shifted to high fields (69.35 ppm for **Ag_29_–1D**, 70.11 ppm for **Ag_29_–2D** and 70.30 ppm for **Ag_29_–3D**) when the Cs^+^ cations were captured by the Ag_29_(SSR)_12_ framework. The different ^133^Cs NMR signals of Cs@Ag_29_ nanoclusters originated in the distinct cluster–Cs–solvents interactions of these Ag_29_-based assemblies. Besides, the intense ^31^P NMR signal of **Ag_29_–0D** at 26.20 ppm disappeared after the PPh_3_ ligands were dissociated from the nanocluster surface (Fig. S8B); that is, no phosphine signal was observed in the ^31^P NMR of Cs@Ag_29_ nanoclusters.

**Figure 4.**
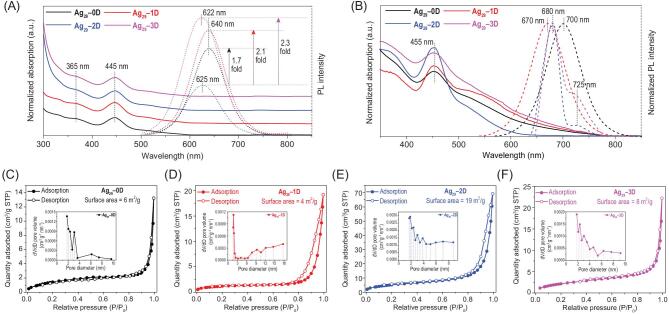
Characterizations of the Ag_29_-based assemblies. (A) Comparison of optical absorptions and emissions (**Ag_29_–0D** and **Ag_29_–1D** were dissolved in DMF; **Ag_29_–2D** was dissolved in NMP; **Ag_29_–3D** was dissolved in TMS) of **Ag_29_–0D** (black lines), **Ag_29_–1D** (red lines), **Ag_29_–2D** (blue lines) and **Ag_29_–3D** (magenta lines) nanoclusters. (B) Comparison of optical absorptions and emissions (nanoclusters were in a crystallized film) of **Ag_29_–0D** (black lines), **Ag_29_–1D** (red lines), **Ag_29_–2D** (blue lines) and **Ag_29_–3D** (magenta lines) nanoclusters. (C–F) Nitrogen adsorption–desorption isotherm and the corresponding pore-size distribution of (C) **Ag_29_–0D**, (D) **Ag_29_–1D**, (E) **Ag_29_–2D** and (F) **Ag_29_–3D** nanoclusters, respectively. Insets: pore size distributions of different Ag_29_-based assemblies.

The structures of nanoclusters are determinant of their physical-chemical properties. Due to their distinct surface structures and crystalline packing modes, these Ag_29_-based assemblies manifested distinguishable optical absorptions and emissions in both solutions and crystallized films. Of note, the solution-state UV-vis and photoluminescence (PL) spectra of **Ag_29_–0D** and **Ag_29_–1D** were monitored in DMF, whereas **Ag_29_–2D** was in NMP and **Ag_29_–3D** was in TMS. The optical absorptions of these Ag_29_ nanoclusters in the solution state were very similar (Fig. 4A, solid lines)―an intense peak at 445 nm and a shoulder band at 365 nm. Such a similarity might result from the fact that the molecularly electronic transitions of these nanoclusters mainly originated in their almost identically inner Ag_29_(SSR)_12_ framework. For the PL, all nanocluster solutions emitted when illuminated at 445 nm (Fig. 4A, dotted lines); however, remarkable differences took place. The DMF solutions of both **Ag_29_–0D** and **Ag_29_–1D** emitted at 640 nm, whereas the emission wavelengths of **Ag_29_–2D** (in NMP) and **Ag_29_–3D** (in TMS) exhibited obvious blue-shifts, of which **Ag_29_–2D** emitted at 625 nm and **Ag_29_–3D** luminesced at 622 nm. Furthermore, the PL

intensities of **Ag_29_–0D**, **Ag_29_–1D** and **Ag_29_–3D** showed 1.7-, 2.1- and 2.3-fold enhancement, respectively, relative to that of **Ag_29_–2D** with the lowest PL intensity. These differences reflected both the structural effect and the solvent effect on nanocluster emissions.

The nanocluster crystallized films exhibited apparent differences in both optical absorptions and emissions (Fig. 4B). The UV-vis spectrum of each nanocluster presented an intense absorption at 455 nm; however, the features of these spectra varied greatly―the 455 nm signal of **Ag_29_–2D** was much more intense than those of other nanoclusters, and the UV-vis spectrum of **Ag_29_–1D** showed a broad shoulder band at 550 nm that was absent for other nanoclusters (Fig. 4B, solid lines). The normalized emissions of these Ag_29_ nanoclusters in crystallized films were further compared. Both **Ag_29_–0D** and **Ag_29_–1D** films were singly emissive: the former emitted at 700 nm and the latter emitted at 670 nm. Of note, the **Ag_29_–0D** film emitted at 700 nm when crystallized in the cubic unit cell, or at 670 nm when crystallized in the trigonal unit cell [50]. By comparison, both **Ag_29_–2D** and **Ag_29_–3D** films were dual-emissive: although the two of them luminesced at 680 and 725 nm, the shoulder emission (725 nm) of **Ag_29_–3D** was more distinguishable than that of the **Ag_29_–2D** (Fig. 4B, dotted lines). The conspicuous differences in emissions of these Ag_29_-based assemblies in different forms (crystal film and solution) arose from distinct combinations of the electronic coupling and the lattice-origin, non-radiative decay pathways occurring through electron–phonon interactions [49,52,53]. Besides, these differences can also be explained in terms of the diverse surface chemistry of these nanoclusters: the PPh_3_ ligand surface of **Ag_29_–0D**, and the distinct cluster–Cs–solvents surfaces of **Ag_29_–1D**, **Ag_29_–2D** and **Ag_29_–3D**.

Because of their different crystalline packing modes, these Ag_29_-based assemblies should exhibit distinctive surface areas. Herein, the nitrogen adsorption–desorption tests were performed on the crystals of these Ag_29_ nanoclusters for evaluating their specific surface area and pore size distribution (Figs 4C–F and S9). The values of the specific surface areas of **Ag_29_–0D**, **Ag_29_–1D** and **Ag_29_–3D** were all below 10 m^2^/g (about 6, 4 and 8 m^2^/g for **Ag_29_–0D**, **Ag_29_–1D** and **Ag_29_–3D**, respectively). By comparison, the **Ag_29_–2D** crystal generated a much bigger specific surface area of about 19 m^2^/g. In this context, as to this Ag_29_ system, the nanocluster crystals would expose the maximum surface areas when the cluster nano-building blocks were assembled into 2D arrays. Indeed, compared with other cluster crystals, the **Ag_29_–2D** crystal presented larger pore sizes (Fig. 4C–F, insets).

## CONCLUSION

The cluster-based 1D linear chains, 2D grid networks and 3D superstructures were selectively constructed by the self-assembly of Ag_29_(SSR)_12_ nano-building blocks with different solvent-conjoining Cs^+^ cations. In the absence of Cs^+^ cations, the bare Ag atoms on Ag_29_(SSR)_12_ were prone to be stabilized by PPh_3_ ligands, producing the unassembled cluster dots in the crystal lattice. In the presence of Cs^+^ cations, the Ag_29_(SSR)_12_ units could be selectively assembled into distinct arrays with different oxygen-carrying solvents: Cs@Ag_29_(SSR)_12_(DMF)*_x_* as 1D linear chains with the DMF solvent, Cs@Ag_29_(SSR)_12_(NMP)*_x_* as 2D grid networks with the NMP solvent, and Cs@Ag_29_(SSR)_12_(TMS)*_x_* as 3D superstructures with the TMS solvent. Besides, the 1D–3D self-assemblies of these Ag_29_(SSR)_12_ nano-building blocks have not only been observed in their crystalline state, but also in their amorphous state, with the help of the aberration-corrected HAADF-STEM. Such Ag_29_-based assemblies manifested distinguishable optical absorptions and emissions in both solutions and crystallized films, and these differences originated from their different surface structures and crystalline packing modes. The surface areas of these Ag_29_ crystals were evaluated, and the 2D-array assembled nanocluster (i.e. Ag_29_-based grid networks) displayed the maximum value of the surface area. Overall, this work presents the hierarchical assembly of atomically precise nanoclusters by simply controlling the adsorbed molecules on the cluster surface, which hopefully sheds light on more future works touching upon the supramolecular chemistry of metal nanoclusters.

## METHODS

### Materials

All reagents were purchased from Sigma-Aldrich and used without further purification: silver nitrate (AgNO_3_, 99%, metal basis), triphenylphosphine (PPh_3_, 99%), 1,3-benzene dithiol (SSR, 99%), sodium borohydride (NaBH_4_, 99.9%), cesium acetate (CH_3_COOCs, 99%), methylene chloride (CH_2_Cl_2_, HPLC, Sigma-Aldrich), methanol (CH_3_OH, HPLC, Sigma-Aldrich), N, N-dimethylformamide (DMF, HPLC, Sigma-Aldrich), N-methyl-2-pyrrolidone (NMP, HPLC, Sigma-Aldrich), tetramethylene sulfone (TMS, HPLC, Sigma-Aldrich), and ethyl ether ((C_2_H_5_)_2_O, HPLC, Sigma-Aldrich).

### Syntheses and crystallization

#### Synthesis of [Ag_29_(SSR)_12_(PPh_3_)_4_]^3−^ (**Ag_29_–0D**)

The preparation of **Ag_29_–0D** was based on the reported method of the Bakr group [49, 50].

#### Synthesis of Cs@Ag_29_(SSR)_12_(DMF)*_x_* (**Ag_29_–1D**)

The preparation of **Ag_29_–1D** was based on the reported method of the Zhu group [48].

#### Synthesis of Cs@Ag_29_(SSR)_12_(NMP)*_x_* (**Ag_29_–2D**)

The 50-mg **Ag_29_–1D** crystal was dissolved in 5 mL of NMP under vigorous stirring. This NMP solution was poured into 200 mL of CH_2_Cl_2_, and the precipitate was collected and further dissolved in 5 mL of NMP, producing the **Ag_29_–2D** nanocluster. The yield was 95% based on the Ag element (calculated from **Ag_29_–1D**). This NMP solution of **Ag_29_–2D** was directly used for the crystallization and the characterization.

#### Synthesis of Cs@Ag_29_(SSR)_12_(TMS)*_x_* (Ag_29_-3D)

The 50-mg **Ag_29_–1D** crystal was dissolved in 5 mL of TMS under vigorous stirring. This TMS solution was poured into 200 mL of CH_2_Cl_2_, and the precipitate was collected and further dissolved in 5 mL of TMS, producing the **Ag_29_–3D** nanocluster. The yield was 95% based on the Ag element (calculated from **Ag_29_–1D**). This TMS solution of **Ag_29_–3D** was directly used for the crystallization and the characterization.

#### Crystallization of **Ag_29_–2D** and **Ag_29_–3D**

Single crystals of **Ag_29_–0D** and **Ag_29_–1D** were cultivated based on the reported methods [48,49]. Single crystals of **Ag_29_–2D** and **Ag_29_–3D** were cultivated at room temperature by diffusing methanol into the NMP solution of **Ag_29_–2D**, or the TMS solution of **Ag_29_–3D**. After two weeks, red crystals were collected, and the structure of **Ag_29_–2D** or **Ag_29_–3D** was determined. The CCDC numbers of **Ag_29_–2D** and **Ag_29_–3D** are 1961389 and 1941329, respectively.

### Characterization

All UV-vis absorption spectra of nanoclusters were recorded using an Agilent 8453 diode array spectrometer. PL spectra were measured on a FL-4500 spectrofluorometer with the same optical density of 0.1. ESI-MS measurements were performed by MicrOTOF-QIII highresolution mass spectrometer. The sample was directly infused into the chamber at 5 μL/min. For preparing the ESI samples, nanoclusters were dissolved in DMF/NMP/TMS (1 mg/mL) and diluted (*v*/*v* = 1:2) by methanol. ^133^Cs and ^31^P NMR spectra were acquired using a Bruker 600 Avance III spectrometer equipped with a Bruker BBO multinuclear probe (BrukerBioSpin, Rheinstetten, Germany). The Ag_29_-based assemblies were imaged with an aberration-corrected HAADF-STEM technique after the solvent that contained Ag_29_-based assemblies was dropped casting onto ultrathin carbon film TEM grids. The microscope employed was a FEI Themis Z. The electron beam energy was 200 kV. The collecting angle HAADF detector was used to collect signals scattered between 52 (inner angle) and 200 (outer angle) mrad (camera length of 146 mm). The aberration-corrected HAADF-STEM image was obtained by Thermo Scientific Velox software using 1024^*^1024 pixels and dwell time was set to 10 us.

#### Nitrogen adsorption–desorption test

The specific surface area and pore size distribution were calculated from each corresponding nitrogen adsorption–desorption isotherm by applying the Brunauer-Emmett-Teller (BET) equation on ASAP2020 M plus Physisorption. By using the quenched solid density functional theory (QSDFT), the pore size distributions were derived from the sorption data. The BET surface areas of **Ag_29_–0D**, **Ag_20_–1D**, **Ag_29_–2D** and **Ag_29_–3D** samples are about 6, 4, 19 and 8 m^2^/g, respectively. Of note, the experimental errors of the nitrogen adsorption–desorption data might be 5%–10%; however, these errors have no effect on the conclusion that **Ag_29_–2D** displays the maximum value of the surface area because the BET surface area of **Ag_29_–2D** (19 m^2^/g) is remarkably higher than those of the **Ag_29_–0D**, **Ag_20_–1D** and **Ag_29_–3D** samples.

#### Single-crystal analysis

The data collection for single crystal X-ray diffraction was carried out on Stoe Stadivari diffractometer under nitrogen flow, using graphite-monochromatized Cu Kα radiation (λ = 1.54186 Å). Data reductions and absorption corrections were performed using the SAINT and SADABS programs, respectively. The electron density was squeezed by Platon. The structure was solved by direct methods and refined with full-matrix least squares on F^2^ using the SHELXTL software package. All non-hydrogen atoms were refined anisotropically, and all the hydrogen atoms were set in geometrically calculated positions and refined isotropically using a riding model.

## Supplementary Material

nwaa077_Supplemental_Files
