# Supplementary material for: Hierarchical structural complexity in atomically precise nanocluster frameworks
Source: Natl Sci Rev. 2020 Apr 24;8(3):nwaa077. doi: 10.1093/nsr/nwaa077 (PMC8288395; doi:10.1093/nsr/nwaa077)

checkCIF/PLATON report

You have not supplied any structure factors. As a result the full set of tests cannot be run.

THIS REPORT IS FOR GUIDANCE ONLY. IF USED AS PART OF A REVIEW PROCEDURE FOR PUBLICATION, IT SHOULD NOT REPLACE THE EXPERTISE OF AN EXPERIENCED CRYSTALLOGRAPHIC REFEREE.

No syntax errors found. CIF dictionary Interpreting this report

Datablock: 1

```
Bond precision:  C-C = 0.0284 A          Wavelength=1.54186

Cell:               a=35.81100       b=28.25500       c=29.24400
                    alpha=90         beta=90          gamma=90
Temperature:        120 K
```

	Calculated	Reported
Volume	29590.242	29591
Space group	P b c n	P b c n
Hall group	-P 2n 2ab	-P 2n 2ab
Moiety formula	C90.20 H80.76 Ag29 Cs2.64 N3.64 O3.64 S24, 0.36(Cs) [+ solvent]	C90.2 H80.76 Ag29 Cs3 N3.64 O3.64 S24
Sum formula	C90.20 H80.76 Ag29 Cs3 N3.64 O3.64 S24 [+ solvent]	C90.20 H80.76 Ag29 Cs3 N3.64 O3.64 S24
Mr	5570.31	5570.34
Dx,g cm-3	2.501	2.501
Z	8	8
Mu (mm-1)	39.120	39.120
F000	20708.5	20708.0
F000’	20839.39	
h,k,lmax	43,34,35	42,33,35
Nref	27881	26612
Tmin,Tmax	0.050,0.457	0.047,0.603
Tmin’	0.008	

```
Correction method= # Reported T Limits: Tmin=0.047 Tmax=0.603
AbsCorr = MULTI-SCAN

Data completeness= 0.954          Theta(max)= 69.637

R(reflections)= 0.0726( 21688)    wR2(reflections)= 0.1566( 26612)

S = 1.115                   Npar= 1418
```

The following ALERTS were generated. Each ALERT has the format
test-name_ALERT_alert-type_alert-level.
Click on the hyperlinks for more details of the test.

Alert level B

```
PLAT342_ALERT_3_B Low Bond Precision on  C-C Bonds ...............     0.0284 Ang.
```

Alert level C

```
PLAT029_ALERT_3_C _diffrn_measured_fraction_theta_full value Low .      0.970 Why?
PLAT141_ALERT_4_C s.u. on a - Axis Small or Missing .............    0.00000 Ang.
PLAT142_ALERT_4_C s.u. on b - Axis Small or Missing .............    0.00000 Ang.
PLAT143_ALERT_4_C s.u. on c - Axis Small or Missing .............    0.00000 Ang.
PLAT151_ALERT_1_C No s.u. (esd) Given on Volume .................     Please Do !
PLAT220_ALERT_2_C Non-Solvent  Resd 1  C   Ueq(max)/Ueq(min) Range        3.7 Ratio
PLAT222_ALERT_3_C Non-Solv.  Resd 1  H   Uiso(max)/Uiso(min) Range        4.5 Ratio
PLAT241_ALERT_2_C High   'MainMol' Ueq as Compared to Neighbors of       O03M Check
PLAT242_ALERT_2_C Low    'MainMol' Ueq as Compared to Neighbors of       Cs0S Check
PLAT260_ALERT_2_C Large Average Ueq of Residue Including       Cs3      0.238 Check
PLAT411_ALERT_2_C Short Inter H...H Contact  H03K     ..H03K     .       2.07 Ang.
                                                 1-x,y,3/2-z  =      3_656 Check
```

Alert level G

```
PLAT002_ALERT_2_G Number of Distance or Angle Restraints on AtSite         20 Note
PLAT003_ALERT_2_G Number of Uiso or Uij Restrained non-H Atoms ...         96 Report
PLAT004_ALERT_5_G Polymeric Structure Found with Maximum Dimension          2 Info
PLAT042_ALERT_1_G Calc. and Reported MoietyFormula Strings  Differ     Please Check
PLAT068_ALERT_1_G Reported F000 Differs from Calcd (or Missing)...     Please Check
PLAT083_ALERT_2_G SHELXL Second Parameter in WGHT  Unusually Large    1586.05 Why ?
PLAT172_ALERT_4_G The CIF-Embedded .res File Contains DFIX Records         20 Report
PLAT178_ALERT_4_G The CIF-Embedded .res File Contains SIMU Records          4 Report
PLAT300_ALERT_4_G Atom Site Occupancy of Cs00       Constrained at       0.64 Check
PLAT300_ALERT_4_G Atom Site Occupancy of O02Z       Constrained at       0.64 Check
PLAT300_ALERT_4_G Atom Site Occupancy of N13        Constrained at       0.64 Check
PLAT300_ALERT_4_G Atom Site Occupancy of C3         Constrained at       0.64 Check
PLAT300_ALERT_4_G Atom Site Occupancy of C8         Constrained at       0.64 Check
PLAT300_ALERT_4_G Atom Site Occupancy of C9         Constrained at       0.64 Check
PLAT300_ALERT_4_G Atom Site Occupancy of C20        Constrained at       0.64 Check
PLAT300_ALERT_4_G Atom Site Occupancy of C1AA       Constrained at       0.64 Check
PLAT300_ALERT_4_G Atom Site Occupancy of H3A        Constrained at       0.64 Check
PLAT300_ALERT_4_G Atom Site Occupancy of H3B        Constrained at       0.64 Check
PLAT300_ALERT_4_G Atom Site Occupancy of H8A        Constrained at       0.64 Check
PLAT300_ALERT_4_G Atom Site Occupancy of H8B        Constrained at       0.64 Check
PLAT300_ALERT_4_G Atom Site Occupancy of H9A        Constrained at       0.64 Check
PLAT300_ALERT_4_G Atom Site Occupancy of H9B        Constrained at       0.64 Check
PLAT300_ALERT_4_G Atom Site Occupancy of H9C        Constrained at       0.64 Check
PLAT300_ALERT_4_G Atom Site Occupancy of H20A       Constrained at       0.64 Check
PLAT300_ALERT_4_G Atom Site Occupancy of H20B       Constrained at       0.64 Check
PLAT300_ALERT_4_G Atom Site Occupancy of Cs3        Constrained at       0.36 Check
PLAT301_ALERT_3_G Main Residue  Disorder ..............(Resd  1  )         3% Note
PLAT302_ALERT_4_G Anion/Solvent/Minor-Residue Disorder (Resd  2  )       100% Note
PLAT343_ALERT_2_G Unusual          Angle Range in Main Residue for       C03B Check
PLAT606_ALERT_4_G VERY LARGE Solvent Accessible VOID(S) in Structure        ! Info
PLAT720_ALERT_4_G Number of Unusual/Non-Standard Labels ..........        185 Note
PLAT764_ALERT_4_G Overcomplete CIF Bond List Detected (Rep/Expd) .       1.15 Ratio
PLAT774_ALERT_1_G Suspect X-Y Bond in CIF: Ag0E     --Cs0S      ..       4.15 Ang.
PLAT774_ALERT_1_G Suspect X-Y Bond in CIF: Ag0K     --Cs0V      ..       4.16 Ang.
PLAT774_ALERT_1_G Suspect X-Y Bond in CIF: Ag0U     --Cs3       ..       4.56 Ang.
```

```
PLAT774_ALERT_1_G Suspect X-Y Bond in CIF: Ag0U     --Cs3       ..       4.52 Ang.
PLAT774_ALERT_1_G Suspect X-Y Bond in CIF: Cs0V     --Ag0K      ..       4.16 Ang.
PLAT774_ALERT_1_G Suspect X-Y Bond in CIF: S01B     --Cs3       ..       4.06 Ang.
PLAT779_ALERT_4_G Suspect or Irrelevant (Bond) Angle(s) in CIF . #       1170 Check
              O02M -C03F -CS0S    1.555   1.555   1.555             30.90 Deg.
PLAT779_ALERT_4_G Suspect or Irrelevant (Bond) Angle(s) in CIF . #       1196 Check
              O02T -C47  -CS0S    1.555   1.555   1.555             42.20 Deg.
PLAT779_ALERT_4_G Suspect or Irrelevant (Bond) Angle(s) in CIF . #       1223 Check
              O02Z -C1AA -CS00    1.555   1.555   1.555             42.80 Deg.
PLAT780_ALERT_1_G Coordinates do not Form a Properly Connected Set     Please Do !
PLAT860_ALERT_3_G Number of Least-Squares Restraints ............       1916 Note
PLAT868_ALERT_4_G ALERTS Due to the Use of _smtbx_masks Suppressed          ! Info
```

```
   0 ALERT level A = Most likely a serious problem - resolve or explain
   1 ALERT level B = A potentially serious problem, consider carefully
  11 ALERT level C = Check. Ensure it is not caused by an omission or oversight
  44 ALERT level G = General information/check it is not something unexpected

  10 ALERT type 1 CIF construction/syntax error, inconsistent or missing data
   9 ALERT type 2 Indicator that the structure model may be wrong or deficient
   5 ALERT type 3 Indicator that the structure quality may be low
  31 ALERT type 4 Improvement, methodology, query or suggestion
   1 ALERT type 5 Informative message, check
```

It is advisable to attempt to resolve as many as possible of the alerts in all categories. Often the minor alerts point to easily fixed oversights, errors and omissions in your CIF or refinement strategy, so attention to these fine details can be worthwhile. In order to resolve some of the more serious problems it may be necessary to carry out additional measurements or structure refinements. However, the purpose of your study may justify the reported deviations and the more serious of these should normally be commented upon in the discussion or experimental section of a paper or in the "special_details" fields of the CIF. checkCIF was carefully designed to identify outliers and unusual parameters, but every test has its limitations and alerts that are not important in a particular case may appear. Conversely, the absence of alerts does not guarantee there are no aspects of the results needing attention. It is up to the individual to critically assess their own results and, if necessary, seek expert advice.

Publication of your CIF in IUCr journals

A basic structural check has been run on your CIF. These basic checks will be run on all CIFs submitted for publication in IUCr journals (*Acta Crystallographica*, *Journal of Applied Crystallography*, *Journal of Synchrotron Radiation*); however, if you intend to submit to *Acta Crystallographica Section C* or *E* or *IUCrData*, you should make sure that full publication checks are run on the final version of your CIF prior to submission.

Publication of your CIF in other journals

Please refer to the *Notes for Authors* of the relevant journal for any special instructions relating to CIF submission.

PLATON version of 07/08/2019; check.def file version of 30/07/2019

Datablock 1 - ellipsoid plot

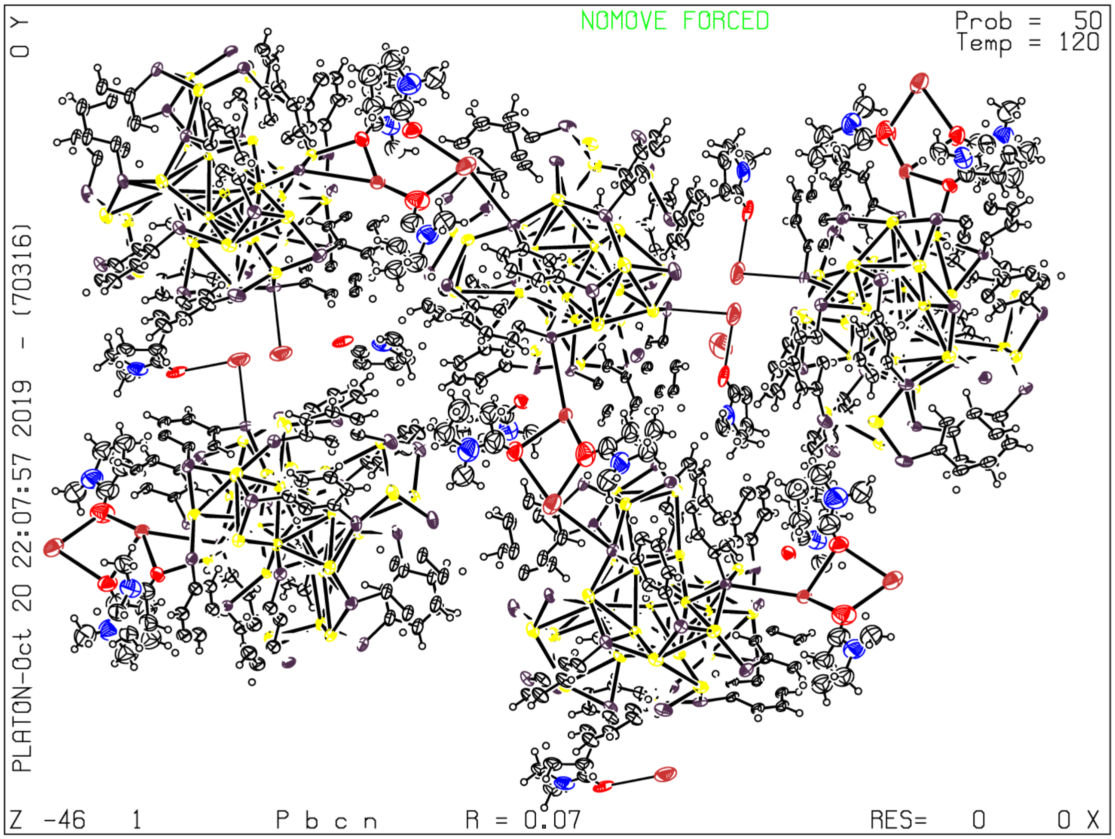

Supplement: nwaa077_Supplemental_Files [file nwaa077_supplemental_files.zip › Ag29-Cs-NMP-2D-Checkcif.pdf]
