# Supplementary material for: Hierarchical structural complexity in atomically precise nanocluster frameworks
Source: Natl Sci Rev. 2020 Apr 24;8(3):nwaa077. doi: 10.1093/nsr/nwaa077 (PMC8288395; doi:10.1093/nsr/nwaa077)

checkCIF/PLATON report

You have not supplied any structure factors. As a result the full set of tests cannot be run.

THIS REPORT IS FOR GUIDANCE ONLY. IF USED AS PART OF A REVIEW PROCEDURE FOR PUBLICATION, IT SHOULD NOT REPLACE THE EXPERTISE OF AN EXPERIENCED CRYSTALLOGRAPHIC REFEREE.

No syntax errors found.   CIF dictionary   Interpreting this report

Datablock: 1

```
Bond precision:  C-C = 0.0411 A          Wavelength=1.54186

Cell:           a=24.3145(15)     b=24.3145(15)     c=24.3145(15)
                alpha=90          beta=90           gamma=90
Temperature:    120 K
```

	Calculated	Reported
Volume	14375(3)	14375(3)
Space group	P 21 3	P 21 3
Hall group	P 2ac 2ab 3	P 2ac 2ab 3
Moiety formula	C72 H48 Ag29 S24, C12 H24 Cs O6 S3, Cs [+ solvent]	0.25(C336 H288 Ag116 Cs8 O24 S108)
Sum formula	C84 H72 Ag29 Cs2 O6 S27 [+ solvent]	C84 H72 Ag29 Cs2 O6 S27
Mr	5437.07	5437.14
Dx,g cm-3	2.512	2.512
Z	4	4
Mu (mm-1)	38.717	38.719
F000	10116.0	10116.0
F000’	10187.01	
h,k,lmax	29,29,29	28,29,29
Nref	9156[4916]	9000
Tmin,Tmax	0.052,0.461	0.159,1.000
Tmin’	0.008	

```
Correction method= # Reported T Limits: Tmin=0.159 Tmax=1.000
AbsCorr = MULTI-SCAN

Data completeness= 1.83/0.98      Theta(max)= 70.328

R(reflections)= 0.0645( 6919)     wR2(reflections)= 0.1827( 9000)

S = 1.020                  Npar= 404
```

The following ALERTS were generated. Each ALERT has the format
test-name_ALERT_alert-type_alert-level.
Click on the hyperlinks for more details of the test.

Alert level B

```
PLAT342_ALERT_3_B Low Bond Precision on  C-C Bonds ...............    0.04111 Ang.
```

Alert level C

```
STRVA01_ALERT_4_C           Flack test results are ambiguous.
           From the CIF: _refine_ls_abs_structure_Flack    0.330
           From the CIF: _refine_ls_abs_structure_Flack_su    0.030
PLAT213_ALERT_2_C Atom C3              has ADP max/min Ratio .....        3.4 prolat
PLAT241_ALERT_2_C High   'MainMol' Ueq as Compared to Neighbors of         C3 Check
PLAT250_ALERT_2_C Large U3/U1 Ratio for Average U(i,j) Tensor ....        2.8 Note
PLAT260_ALERT_2_C Large Average Ueq of Residue Including      Cs00      0.186 Check
PLAT360_ALERT_2_C Short  C(sp3)-C(sp3) Bond  C6       - C0AA     .       1.38 Ang.
```

Alert level G

```
PLAT002_ALERT_2_G Number of Distance or Angle Restraints on AtSite          5 Note
PLAT003_ALERT_2_G Number of Uiso or Uij Restrained non-H Atoms ...         20 Report
PLAT033_ALERT_4_G Flack x Value Deviates > 3.0 * sigma from Zero .      0.330 Note
PLAT042_ALERT_1_G Calc. and Reported MoietyFormula Strings  Differ     Please Check
PLAT072_ALERT_2_G SHELXL First  Parameter in WGHT  Unusually Large       0.11 Report
PLAT083_ALERT_2_G SHELXL Second Parameter in WGHT  Unusually Large     143.10 Why ?
PLAT172_ALERT_4_G The CIF-Embedded .res File Contains DFIX Records          3 Report
PLAT178_ALERT_4_G The CIF-Embedded .res File Contains SIMU Records          3 Report
PLAT300_ALERT_4_G Atom Site Occupancy of Cs0A       Constrained at     0.3333 Check
PLAT302_ALERT_4_G Anion/Solvent/Minor-Residue Disorder (Resd  3  )       100% Note
PLAT304_ALERT_4_G Non-Integer Number of Atoms in ...... Resd  3          0.33 Check
PLAT606_ALERT_4_G VERY LARGE Solvent Accessible VOID(S) in Structure        ! Info
PLAT720_ALERT_4_G Number of Unusual/Non-Standard Labels ..........         60 Note
PLAT764_ALERT_4_G Overcomplete CIF Bond List Detected (Rep/Expd) .       1.34 Ratio
PLAT860_ALERT_3_G Number of Least-Squares Restraints ............         117 Note
PLAT868_ALERT_4_G ALERTS Due to the Use of _smtbx_masks Suppressed          ! Info
```

0 **ALERT level A** = Most likely a serious problem - resolve or explain
1 **ALERT level B** = A potentially serious problem, consider carefully
6 **ALERT level C** = Check. Ensure it is not caused by an omission or oversight
16 **ALERT level G** = General information/check it is not something unexpected

1 ALERT type 1 CIF construction/syntax error, inconsistent or missing data
9 ALERT type 2 Indicator that the structure model may be wrong or deficient
2 ALERT type 3 Indicator that the structure quality may be low
11 ALERT type 4 Improvement, methodology, query or suggestion
0 ALERT type 5 Informative message, check

It is advisable to attempt to resolve as many as possible of the alerts in all categories. Often the minor alerts point to easily fixed oversights, errors and omissions in your CIF or refinement strategy, so attention to these fine details can be worthwhile. In order to resolve some of the more serious problems it may be necessary to carry out additional measurements or structure refinements. However, the purpose of your study may justify the reported deviations and the more serious of these should normally be commented upon in the discussion or experimental section of a paper or in the "special_details" fields of the CIF. checkCIF was carefully designed to identify outliers and unusual parameters, but every test has its limitations and alerts that are not important in a particular case may appear. Conversely, the absence of alerts does not guarantee there are no aspects of the results needing attention. It is up to the individual to critically assess their own results and, if necessary, seek expert advice.

Publication of your CIF in IUCr journals

A basic structural check has been run on your CIF. These basic checks will be run on all CIFs submitted for publication in IUCr journals (*Acta Crystallographica*, *Journal of Applied Crystallography*, *Journal of Synchrotron Radiation*); however, if you intend to submit to *Acta Crystallographica Section C* or *E* or *IUCrData*, you should make sure that full publication checks are run on the final version of your CIF prior to submission.

Publication of your CIF in other journals

Please refer to the *Notes for Authors* of the relevant journal for any special instructions relating to CIF submission.

PLATON version of 03/05/2019; check.def file version of 29/04/2019

Datablock 1 - ellipsoid plot

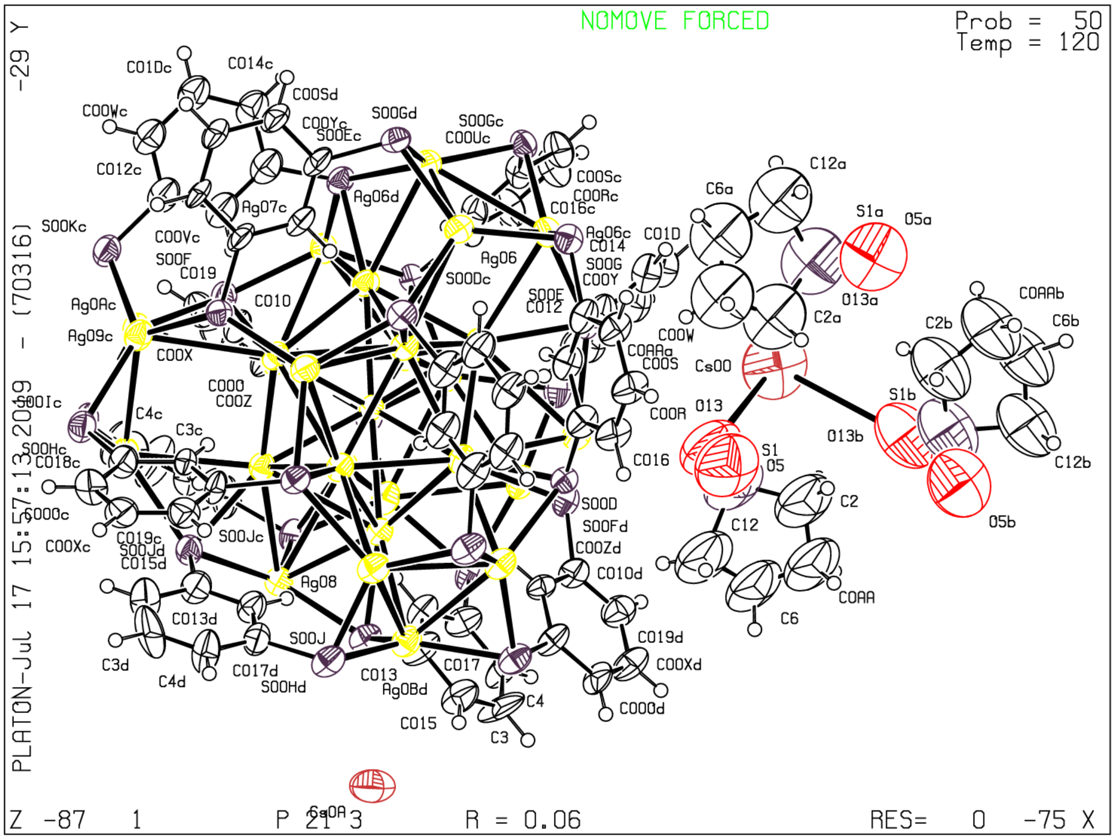

Supplement: nwaa077_Supplemental_Files [file nwaa077_supplemental_files.zip › Ag29-Cs-TMS-3D-Checkcif.pdf]
