# Supplementary material for: Hierarchical structural complexity in atomically precise nanocluster frameworks
Source: Natl Sci Rev. 2020 Apr 24;8(3):nwaa077. doi: 10.1093/nsr/nwaa077 (PMC8288395; doi:10.1093/nsr/nwaa077)
Supplement: nwaa077_Supplemental_Files [file nwaa077_supplemental_files.zip › NSR_MS-2020-126.R1-SI.docx]

Supplementary Information:

Hierarchical Structural Complexity in Atomically Precise Nanocluster Frameworks

Xiao Wei,^1,2,†^ Xi Kang,^1,2,†^ Zewen Zuo,^3,4^ Fengqi Song,^3,4^ Shuxin Wang,^1,2,^* Manzhou Zhu^1,2,^*

**Affiliations**

^1^Department of Chemistry and Centre for Atomic Engineering of Advanced Materials, Anhui Province Key Laboratory of Chemistry for Inorganic/Organic Hybrid Functionalized Materials, Anhui University, Hefei, Anhui, 230601, P. R. China.

^2^Key Laboratory of Structure and Functional Regulation of Hybrid Materials (Anhui University), Ministry of Education, Hefei, 230601, P. R. China.

^3^National Laboratory of Solid State Microstructures, Collaborative Innovation Center of Advanced Microstructures, School of Physics, Nanjing University, Nanjing 210093, P. R. China.

^4^Atomic Manufacture Institute, Nanjing 211805, P. R. China.

^†^X.W. and X.K. contributed equally to this work.

**E-mail**

*To whom correspondence should be addressed.

Email: ixing@ahu.edu.cn (S.W.); zmz@ahu.edu.cn (M.Z.)

**This supplementary file includes:**

Figures. S1 to S9

Tables S1 to S4

**
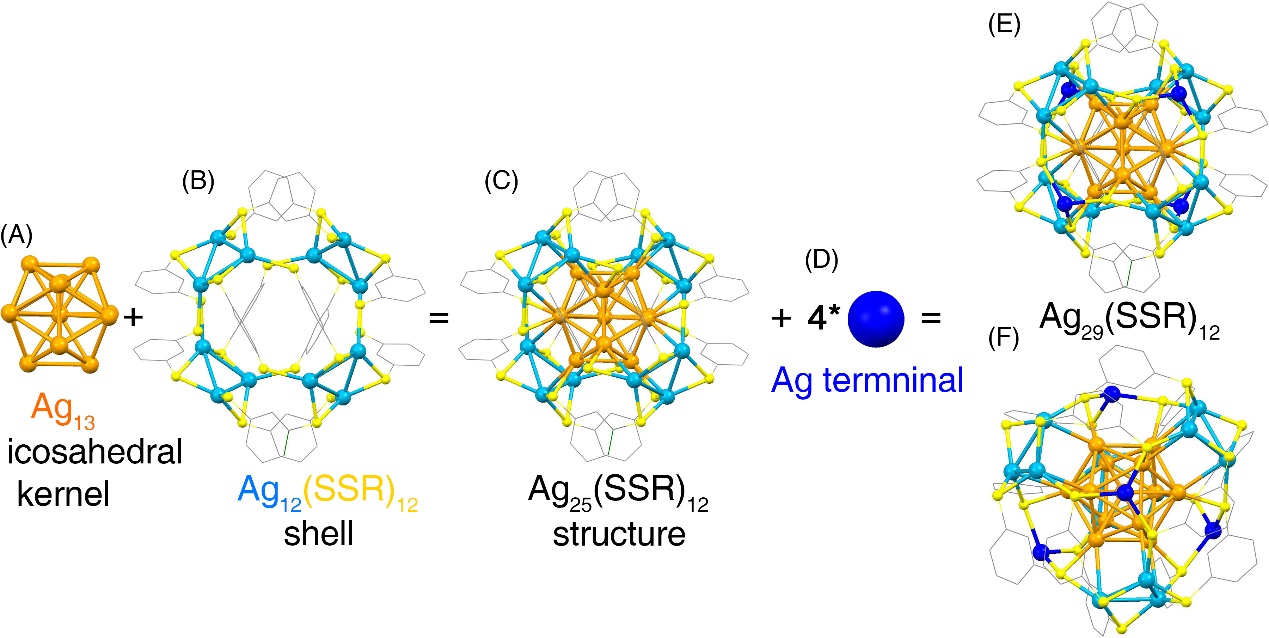
**

**Figure S1.** Structural anatomy of the Ag_29_(SSR)_12_ framework. (A) The icosahedral Ag_13_ kernel. (B) The Ag_12_(SSR)_12_ shell. (C) The Ag_25_(SSR)_12_ structure. (D) The terminal Ag atom. (E,F) The Ag_29_(SSR)_12_ framework, viewed from different angles. Color codes: orange sphere, Ag in the icosahedral kernel; light blue sphere, Ag in the Ag_12_(SSR)_12_ shell; blue sphere, terminal Ag; yellow sphere, S; grey sphere, C. For clarify, the H atoms are omitted.

**
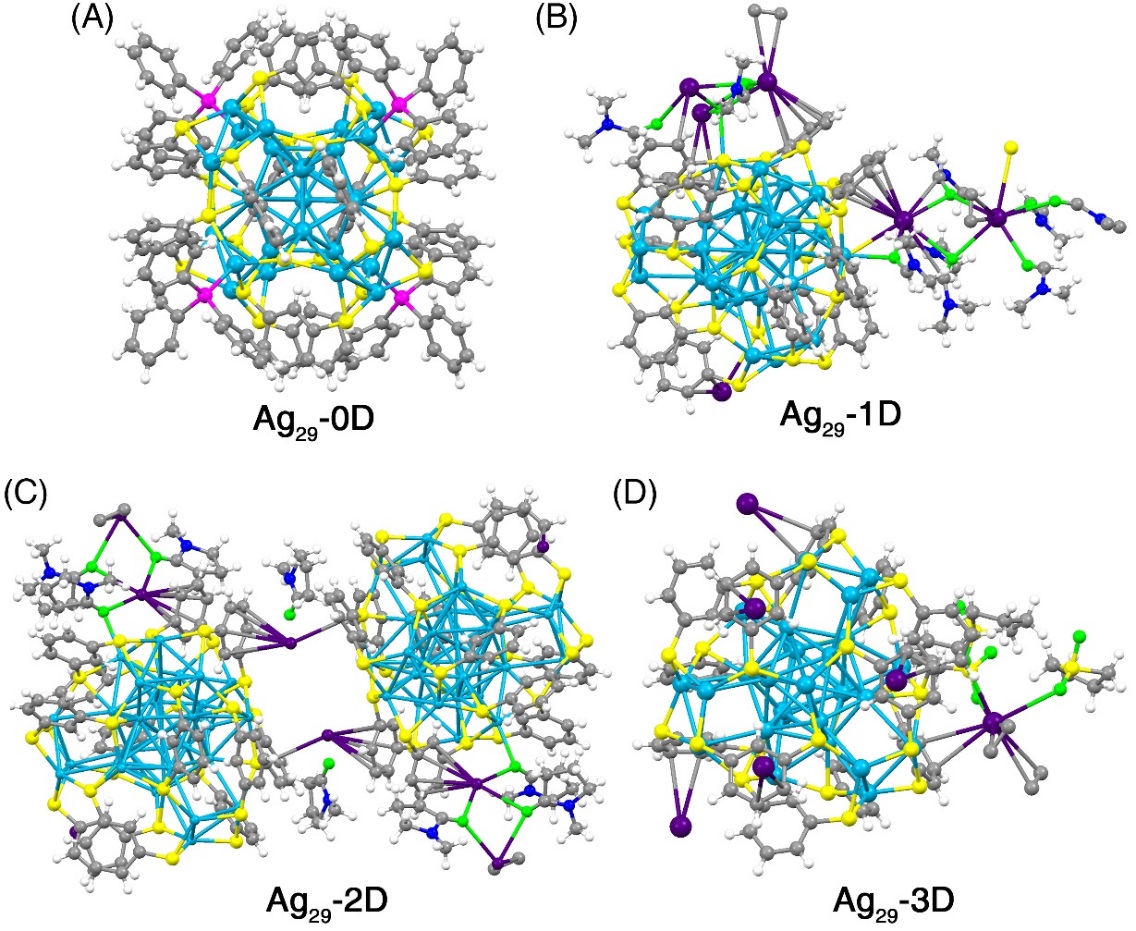
**

**Figure S2.** Overall structures of **Ag_29_-0D**, **Ag_29_-1D**, **Ag_29_-2D**, and **Ag_29_-3D** nanoclusters. Color codes: light blue sphere, Ag; yellow sphere, S; magenta sphere, P; grey sphere, C; white sphere, H; blue sphere, N; green sphere, O; purple sphere, Cs.

**
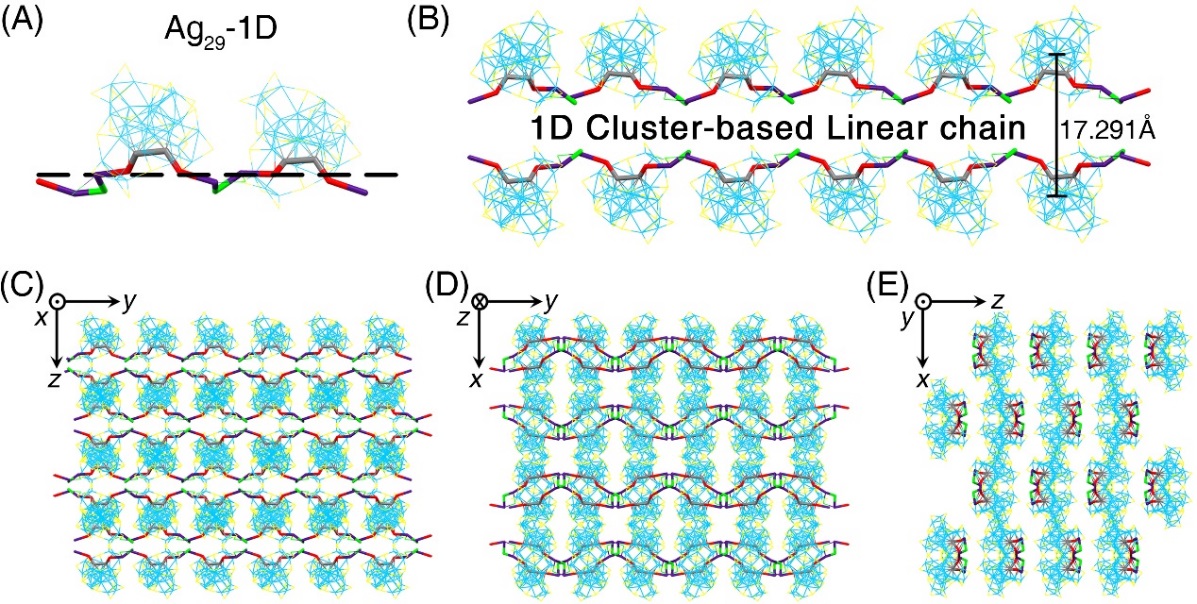
**

**Figure S3.** (A) The cluster unit of **Ag_29_-1D**. The two Ag_29_ nanocluster units are in differently twisting angles. (B) Two adjacent one-dimension **Ag_29_-1D** linear chains. (C-E) Packing of the **Ag_29_-1D** in crystal lattices: view from the *x* axis (C), *z* axis (D), and *y* axis (E). Color legend: light blue/grey sphere, Ag; dark purple sphere, Cs; yellow/red sphere, S; green sphere, O. For clarity, all H atoms, C atoms, N atoms, and some Cs^+^ ions and DMF molecules are omitted. Each O atom represents a DMF molecule.

**
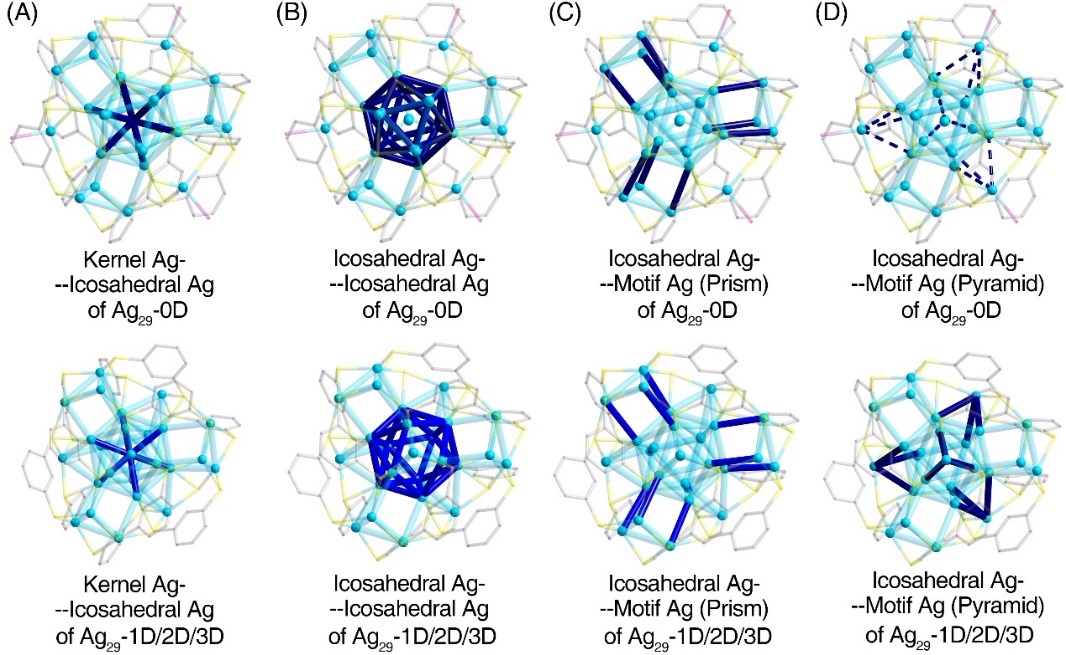
**

**Figure S4.** Comparison of the bond lengths of **Ag_29_-0D**, **Ag_29_-1D**, **Ag_29_-2D**, and **Ag_29_-3D** nanoclusters. (A) Bonds between Ag(core) and Ag(icosahedral shell), highlighted in blue. (B) Bonds between Ag(icosahedral shell) and Ag(icosahedral shell), highlighted in blue. (C) Prism-like bonds between Ag(icosahedral shell) and Ag(motif), highlighted in blue. (C) Pyramid-like bonds between Ag(icosahedral shell) and Ag(motif), highlighted in blue. These bond lengths are further shown in Table S1.

**
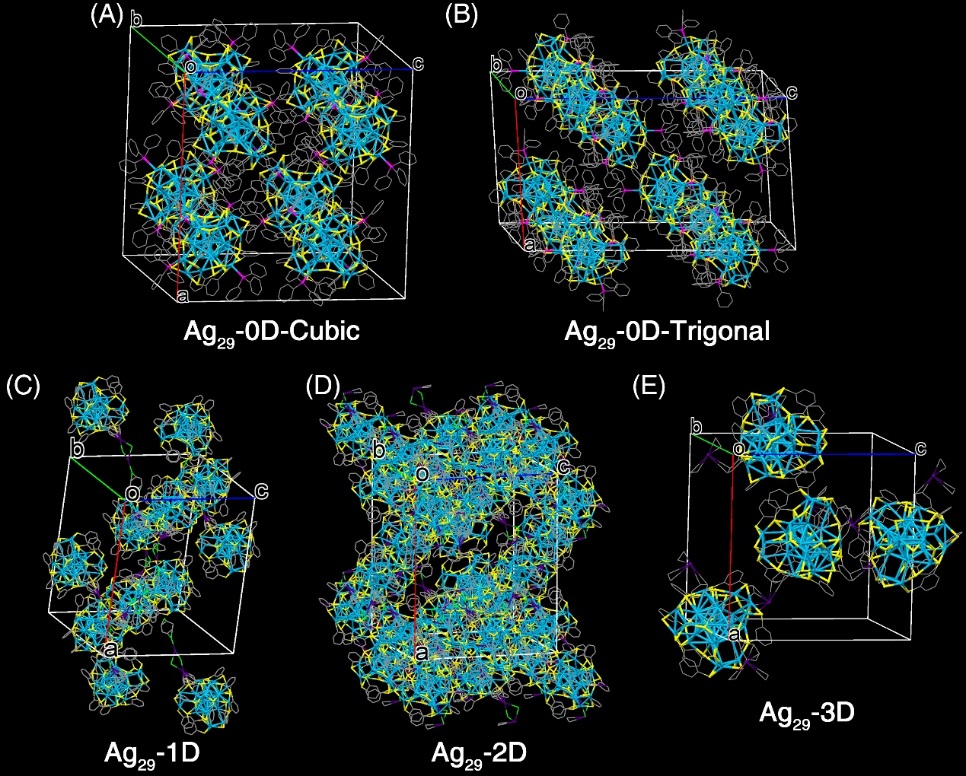
**

**Figure S5.** Crystal lattices of (A,B) **Ag_29_-0D**, (C) **Ag_29_-1D**, (D) **Ag_29_-2D**, and (E) **Ag_29_-3D** nanoclusters. For the **Ag_29_-0D** nanocluster, two types of crystalline packing modes have been reported ― cubic packing and trigonal packing ― due to the different crystallization modes of them. Color codes: light blue sphere, Ag; yellow sphere, S; magenta sphere, P; grey sphere, C; white sphere, H; blue sphere, N; green sphere, O; purple sphere, Cs.

**
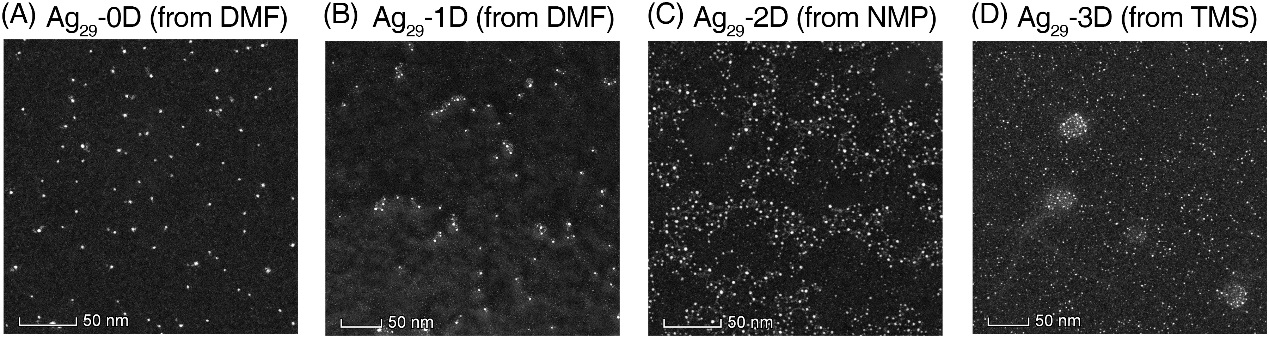
**

**Figure S6.** The aberration-corrected HAADF-STEM images of (A) **Ag_29_-0D**, (B) **Ag_29_-1D**, (C) **Ag_29_-2D**, and (D) **Ag_29_-3D**. Scale bar = 50 nm.

**
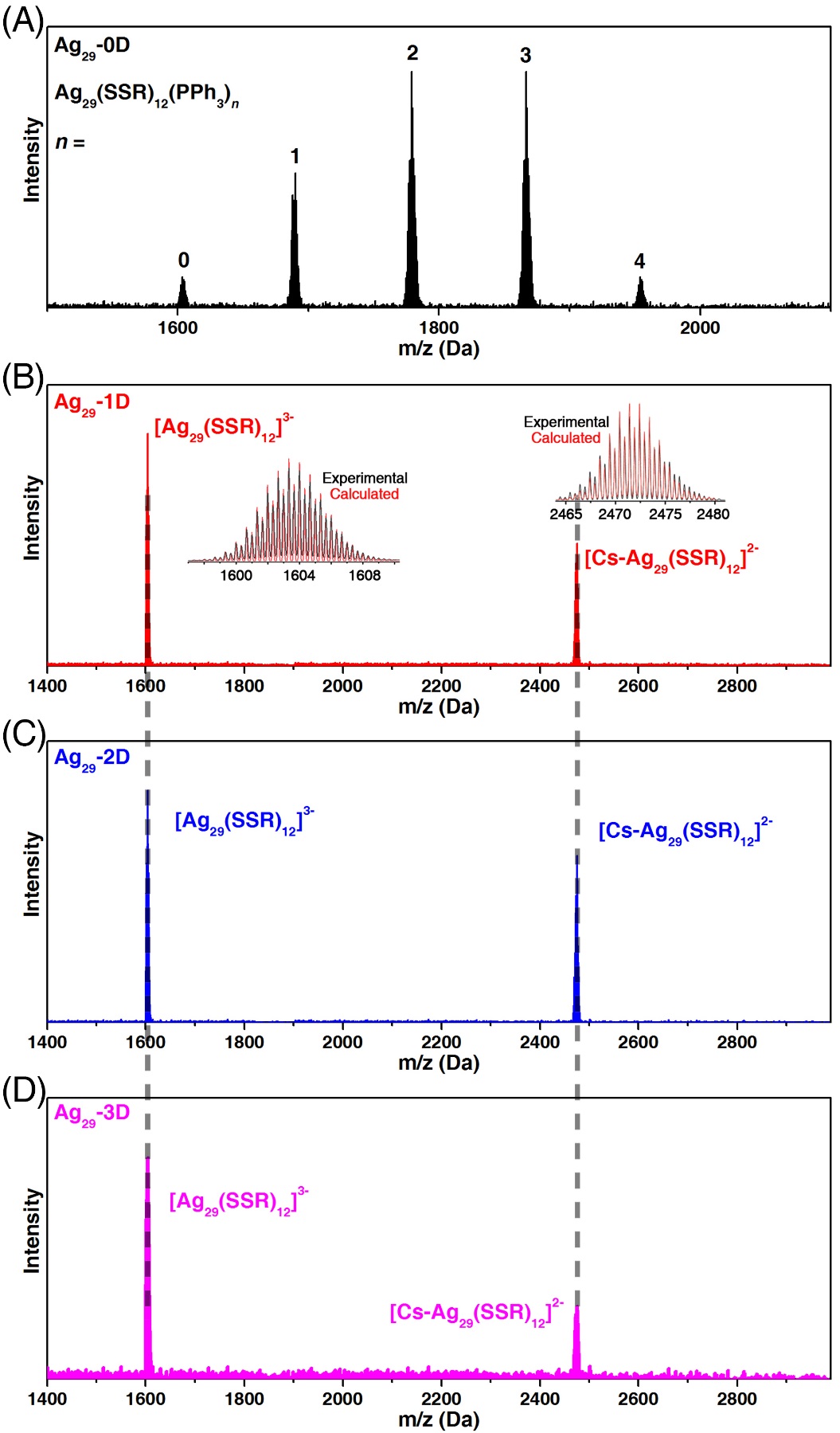
**

**Figure S7.** ESI-MS results of (A) **Ag_29_-0D**, (B) **Ag_29_-1D**, (C) **Ag_29_-2D**, and (D) **Ag_29_-3D** nanoclusters. (A) The five peaks correspond to the [Ag_29_(SSR)_12_(PPh_3_)*_n_*]^3-^ compounds where *n* = 0-4. (B-D) The two peaks in each mass spectrum correspond to [Ag_29_(SSR)_12_]^3-^ and [Cs-Ag_29_(SSR)_12_]^2-^, respectively. Insets in B: the experimental and simulated isotope patterns of [Ag_29_(SSR)_12_]^3-^ and [Cs-Ag_29_(SSR)_12_]^2^.

**
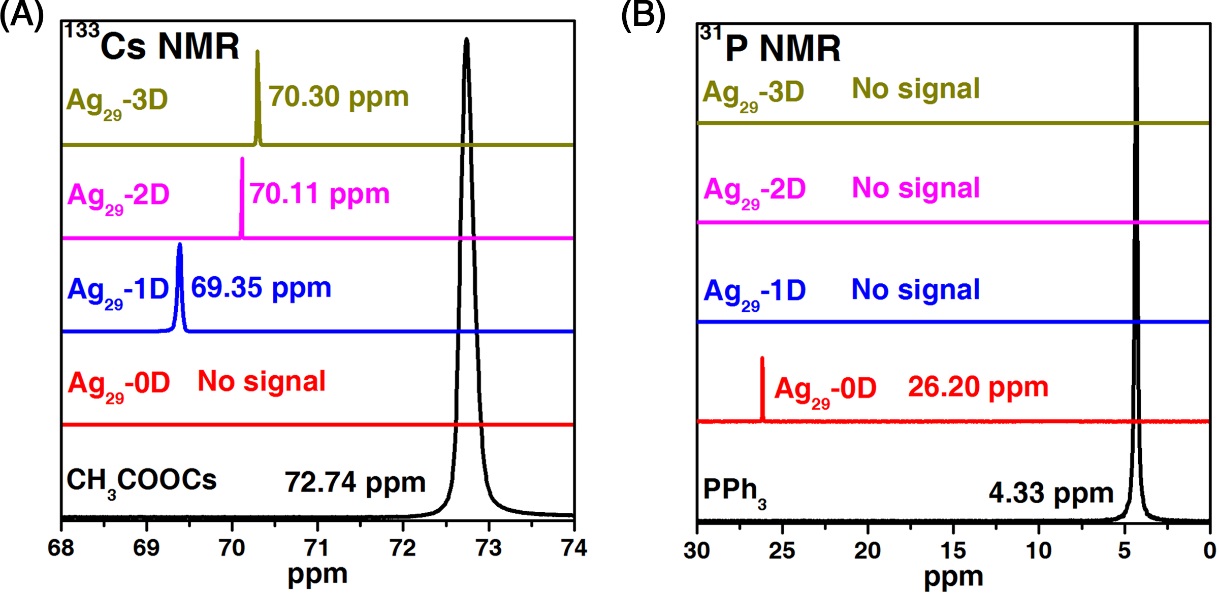
**

**Figure S8.** (A) ^133^Cs NMR spectra of the CH_3_COOCs salt and Ag_29_-based assemblies. (B) ^31^P NMR spectra of the PPh_3_ ligand and Ag_29_-based assemblies.

**
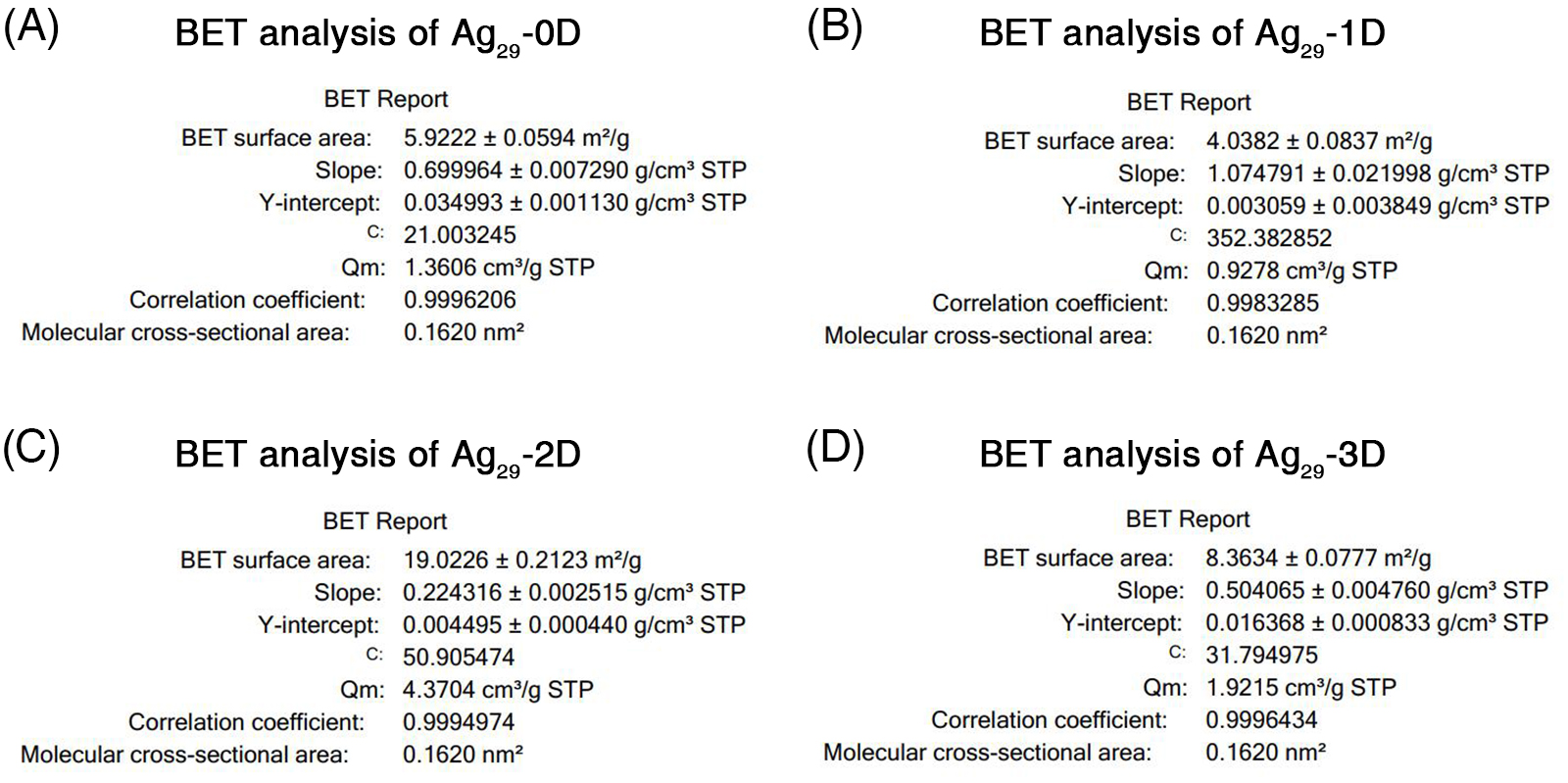
**

**Figure S9.** Comparison of BET data of Ag_29_-based assemblies.

**Table S1.** Comparison of bond lengths of **Ag_29_-0D_,_ Ag_29_-1D, Ag­_29_ –2D and Ag_29_-3D** nanoclusters. These bonds are depicted in Figure S4.

| Cluster | Kernel Ag-Icosahedral Ag | | Icosahedral Ag-Icosahedral Ag | | Icosahedral Ag-Motif Ag (Prism) | | Icosahedral Ag-Motif Ag (Pyramid) | |
| --- | --- | --- | --- | --- | --- | --- | --- | --- |
|  | Range (Å) | Avg.(Å) | Range (Å) | Avg.(Å) | Range (Å) | Avg.(Å) | Range (Å) | Avg.(Å) |
| **Ag_29_-0D** | 2.755-2.772 | 2.765 | 2.834-2.975 | 2.907 | 3.077-3.158 | 3.111 | 3.493-3.643 | 3.523 |
| **Ag_29_-1D** | 2.769-2.803 | 2.788 | 2.800-3.080 | 2.937 | 3.064-3.223 | 3.132 | 2.987-3.106 | 3.043 |
| Diff. | - | +0.83% | - | +1.03% | - | +0.68% | - | 15.77% |
| **Ag_29_-2D** | 2.768-2.810 | 2.788 | 2.800-2.835 | 2.967 | 3.065-3.223 | 3.144 | 2.975-3.085 | 3.039 |
| Diff |  | +0.83% |  | +2.02% |  | +1.06% |  | 13.74% |
| **Ag_29_-3D** | 2.780-2.816 | 2.791 | 2.815-3.065 | 2.927 | 3.082-3.181 | 3.121 | 3.016-3.059 | 3.036 |
| Diff |  | +0.94% |  | +0.69% |  | +0.32% |  | 13.82% |

**Table S2.** Comparison of the unit cell parameters of **Ag_29_-0D_,_ Ag_29_-1D, Ag­_29_ –2D and Ag_29_-3D** nanoclusters.

| Unit Cell | **Ag_29_-0D-cubic** | **Ag_29_-0D-trigonal** | **Ag_29_-1D** | **Ag_29_-2D** | **Ag_29_-3D** |
| --- | --- | --- | --- | --- | --- |
| a, b, c/Å | 34.2011(8), 34.2011(8), 34.2011(8) | 27.4634(6), 27.4634(6), 46.6552(16) | 35.1264(11), 27.9091(10), 29.5657(12) | 35.811, 28.255, 29.2440 | 24.3145(15), 24.3145(15), 24.3145(15) |
| α, β, γ/° | 90, 90, 90 | 90, 90, 120 | 90, 90, 90 | 90, 90, 90 | 90, 90, 90 |
| Volume/Å^3^ | 40006(3) | 30474.7(17) | 28984.6(18) | 29590.5 | 14375(3) |
| Crystal system | Cubic | Trigonal | Orthorhombic | Orthorhombic | Cubic |
| Space group | Pa -3 | R -3 | P b c n | P b c n | P 2_1_ 3 |
| Density (g cm^-3^) | 2.116 | 2.041 | 2.590 | 2.501 | 2.512 |
| Z value | 8 | 6 | 8 | 8 | 4 |

**Table S3**. Crystal data and structure refinement for the **Ag_29_-2D** nanocluster.

| Crystal system | orthorhombic |
| --- | --- |
| Space group | *P b c n* |
| a/Å | 35.811 |
| b/Å | 28.255 |
| c/Å | 29.244 |
| α/° | 90 |
| β/° | 90 |
| γ/° | 90 |
| Volume/Å^3^ | 29590.5 |
| Z | 8 |
| ρ_calc_g/cm^3^ | 2.501 |
| μ/mm^‑1^ | 39.120 |
| F(000) | 20708 |
| Radiation | CuKα (λ = 1.54186) |
| Index ranges | -42 ≤ h ≤ 18, -26 ≤ k ≤ 33, -31 ≤ l ≤ 35 |
| Final R indexes [I>=2σ (I)] | R_1_ = 0.0726, wR_2_ = 0.1501 |
| Final R indexes [all data] | R_1_ = 0.0881, wR_2_ = 0.1566 |

**Table S4**. Crystal data and structure refinement for the **Ag_29_-3D** nanocluster.

| Crystal system | cubic |
| --- | --- |
| Space group | *P* 21 3 |
| a/Å | 24.3145(15) |
| b/Å | 24.3145(15) |
| c/Å | 24.3145(15) |
| α/° | 90 |
| β/° | 90 |
| γ/° | 90 |
| Volume/Å^3^ | 14375(3) |
| Z | 4 |
| ρ_calc_g/cm^3^ | 2.512 |
| μ/mm^‑1^ | 38.719 |
| F(000) | 10116 |
| Radiation | CuKα (λ = 1.54186) |
| Index ranges | -28 ≤ h ≤ 11, -29 ≤ k ≤ 23, -29 ≤ l ≤ 26 |
| Final R indexes [I>=2σ (I)] | R_1_ = 0.0645, wR_2_ = 0.1601 |
| Final R indexes [all data] | R_1_ = 0.0930, wR_2_ = 0.1827 |
